# Comparison of perioperative outcomes of robotic vs. laparoscopic partial nephrectomy for renal tumors with a RENAL nephrometry score ≥7: A meta-analysis

**DOI:** 10.3389/fsurg.2023.1138974

**Published:** 2023-03-15

**Authors:** Yu-Li Jiang, Dong-dong Yu, Yang Xu, Ming-Hua Zhang, Fu-Sheng Peng, Peng Li

**Affiliations:** Department of Urology, Hu Zhou Central Hospital, Huzhou, China

**Keywords:** robotic nephrectomy, laparoscopic partial nephrectomy, renal tumors, RENAL nephrometry score, meta-analysis

## Abstract

**Introduction:**

To compare the perioperative outcomes of robotic partial nephrectomy (RPN) vs. laparoscopic partial nephrectomy (LPN) for complex renal tumors with a RENAL nephrometry score ≥7.

**Methods:**

We searched PubMed, EMBASE and the Cochrane Central Register for studies from 2000 to 2020 to evaluate the perioperative outcomes of RPN and LPN in patients with a RENAL nephrometry score ≥7. We used RevMan 5.2 to pool the data.

**Results:**

Seven studies were acquired in our study. No significant differences were found in the estimated blood loss (WMD: 34.49; 95% CI: −75.16–144.14; *p* = 0.54), hospital stay (WMD: −0.59; 95% CI: −1.24–0.06; *p* = 0.07), positive surgical margin (OR: 0.85; 95% CI: 0.65–1.11; *p* = 0.23), major postoperative complications (OR: 0.90; 95% CI: 0.52–1.54; *p* = 0.69) and transfusion (OR: 0.72; 95% CI: 0.48–1.08; *p* = 0.11) between the groups. RPN showed better outcomes in the operating time (WMD: −22.45; 95% CI: −35.06 to −9.85; *p* = 0.0005), postoperative renal function (WMD: 3.32; 95% CI: 0.73–5.91; *p* = 0.01), warm ischemia time (WMD: −6.96; 95% CI: −7.30–−6.62; *p* < 0.0001), conversion rate to radical nephrectomy (OR: 0.34; 95% CI: 0.17 to 0.66; *p* = 0.002) and intraoperative complications (OR: 0.52; 95% CI: 0.28–0.97; *p* = 0.04).

**Discussion:**

RPN is a safe and effective alternative to LPNs for or the treatment of complex renal tumors with a RENAL nephrometry score ≥7 with a shorter warm ischemic time and better postoperative renal function.

## Introduction

Partial nephrectomy (PN) or nephron-sparing surgery (NSS) is considered the gold standard surgical strategy for clinical T1 renal tumors (1). NSS can demonstrate equivalent oncologic outcomes and better postoperative renal function than radical nephrectomy. In 1993, Gill et al. first introduced laparoscopic partial nephrectomy (LPN) in patients with a single renal tumor (2). LPN has the advantage of reaching similar oncological outcomes, a shorter hospital stay and a lower estimated blood loss. Thus, LPN has been widely used for small renal tumors during the past decades (2–7). Despite the different LPN techniques, several limitations exist, such as the requirement for technically demanding intracorporeal suture reconstruction skills and difficulty of tumor excision (8).

Robot-assisted partial nephrectomy was first reported in 2004 and has become a popular surgical method for clinical T1 renal tumors. The main advantages of the robotic system include the three-dimensional high-definition vision of the surgical field, a great range of wristed instruments, higher precision in the surgical dissection and easier intracorporeal sutured reconstruction, making RPN more popular than LPN (8–12). Recently, Mari et al. conducted a multicenter prospective study evaluating PN for complex renal tumors. They found that PN is safe for complex renal masses and acquired good oncological and functional results (13).

Several meta-analyses had reported two surgical methods for renal tumors (14–16). Aboumarzouk et al. performed a meta-analysis including 717 patients comparing robotic partial nephrectomy (RPN) with laparoscopic partial nephrectomy (LPN). They found that RPN is a safe and feasible option for LPN (14). Choi et al. conducted a meta-analysis including 23 studies involving 2,240 patients and found that RPN shows better recovery of postoperative renal function and a lower conversion rate to radical nephrectomy (16). However, no meta-analysis has been performed to compare RPN and LPN in treating complex renal tumors with a RENAL nephrometry score ≥7. This meta-analysis aimed to evaluate the perioperative, outcomes between RPN and LPN for complex renal tumors with a RENAL nephrometry score ≥7.

## Methods

### Search strategy

We conducted this meta-analysis following the Preferred Reporting Items for Systematic Reviews and Meta-Analysis (PRISMA) guidelines. We searched relevant studies in PubMed, EMBASE and the Cochrane Central Register published in English between 2000 and 2020. We used the following search terms: “robotic partial nephrectomy (RPN) [MeSH]”, “laparoscopic partial nephrectomy (LPN) [MeSH]”, “renal tumor* [MeSH]”, and “RENAL nephrometry score ≥7* OR complex renal tumors *”. We also used the combined Boolean operators “AND” or “OR” in the title/abstract.

### Inclusion and exclusion criteria

**Figure 1:**
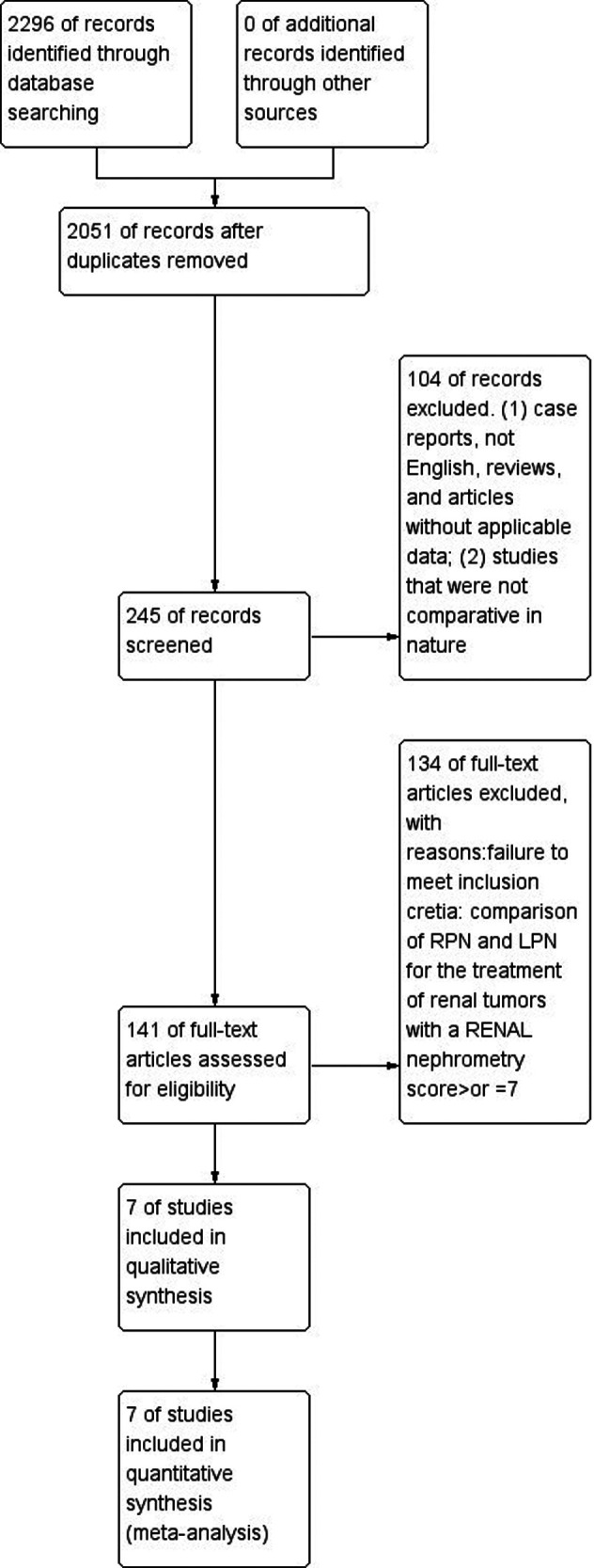
Flow diagram of the process for the selection of relevant studies.

The inclusion and exclusion criteria of studies followed PICOS principles. (1) Participants: patients with renal tumor with a RENAL nephrometry score ≥7. (2) interventions and comparisons: group1 patients received robotic partial nephrectomy, group2 patients received laparoscopic partial nephrectomy; outcomes: estimated blood loss, hospital stay, intraoperative complications, postoperative complications, operative time, conversion rate to radical nephrectomy, positive surgical margin (PSM), transfusion, warm ischemia time, and postoperative renal function; Study design comparative study of RPN and LPN to treat renal tumors with a RENAL nephrometry score ≥7; case reports, reviews, editorial comments, meeting abstracts and articles without applicable data were excluded. The process of identifying relevant studies is summarized in Figure 1. Two investigators (YLJ and DDY) reviewed the articles.

### Data extraction

The two authors extracted data, such as the estimated blood loss, operating time, positive surgical margin, postoperative complications, intraoperative complications, hospital stay, confusion, conversion rate to radical nephrectomy, postoperative renal function and warm ischemia time. The disagreements were resolved by the two reviewers (YLJ and YX). The main outcomes explored within the seven studies (Table 1).

**Table 1:** Outcomes of the included studies.

Study time	PSM		Operating		WIT		Transfusion		Conversion		Postoperative renal function		Major posroperative complications	
					(min)									
	RPN	LPN	RPN	LPN	RPN	LPN	RPN	LPN	RPN	LPN	RPN	LPN	RPN	LPN
			(min)											
Long	1	1	196.9	240	22.4	23.2	24	26	2	21	NA	NA	11	9
Jang	0	1	143.9	168	24.7	27.3	4	4	0	0	NA	NA	1	1
Wang	1	2	135.6	149	20.5	8.4	2	5	2	5	−7	−8.6	3	6
Gu	1	1	NA	NA	NA	NA	6	8	1	2	−5.5	−11.3	4	2
Deng	2	1	198.8	219	NA	NA	5	11	2	4	−7.9	−11.3	3	4
Alimi	1	3	134	146	15.7	23	3	4	3	0	NA	NA	4	5
Zhang	NA	NA	NA	NA	NA	NA	2	3	NA	NA	NA	NA	0	2

*NA*, not avaliable.

### Statistical analysis

We used Review Manager Version 5.2 software and the Mantel-Haenszel method (The Cochrane Collaboration, Oxford, UK) to conduct the data analysis. For quantitative data, we used the standard mean difference (SMD) and 95% confidence interval (CI) or weight mean difference (WMD) and 95% CI to pool continuous data. We used the odds ratio (OR) and 95% CI to calculate binary data. Cochran's Q test was used to evaluate the heterogeneity; *I*^2 ^< 50% or *p* > 0.01 was associated with little heterogeneity. Otherwise, *I*^2 ^> 50% or *p* < 0.01 was related to high heterogeneity. The statistical significance level was 0.05.

### Quality assessment of the included studies

The New-Ottawa Scale (NOS) was used to evaluate the nonrandomized studies (17, 18). The NOS scores were evaluated using a 9-point system. For randomized controlled trials, we assessed the risk of bias according to the Cochrane Collaboration handbook, version 5.0. Table 2 shows the quality assessment of the included studies.

**Table 2:** Newcastle-Ottawa scale for risk of bias assessment of the included studies.

Study	Design	Selection				Comparability	Outcome			Total
		Representativeness of exposed cohort	Selective of nonexposed Cohort	Ascertainment of exposure	Outcome not present at start		Assessment of outcome	Adequate follow-up length	Adequacy of follow-up	
Long	P	**	**		*		*	*	*	8
Jang	R	*	*	*	*		*	*	*	7
Wang	R	*	*	*	*		*	*	*	7
Gu	R	*	*	*	*		*	*	*	7
Deng	R	*	*	*	*		*	*	*	7
Alimi	P	**	**		*		*	*	*	8
Zhang	P	*	*		*		*	*	*	6

*R*, Respective; *P*, Prospective.

## Results

Seven studies were involved in our study (1, 10, 19–23). The literature searching process is summarized in Figure 1. From the PubMed, EMBASE and the Cochrane Central Register, we acquired 2,296 studies. After a precise search, we included 245 studies. After further processing, we excluded 104 studies. Finally, 7 studies meeting the inclusion criteria were included in this meta-analysis. Table 3 summarizes the baseline characteristics and assessments of the included studies.

**Table 3:** Basic characteristics of the included studies.

Study	Year	Design	Sample Size		Mean age(years)		Tumor Size(cm)		BMI (kg/m^2^)		RENAL score (sample size)	
			RPN	LPN	RPN	LPN	RPN	LPN	RPN	LPN	RPN	LPN
Long	2012	P,S	199	182	58.5	59.5	3.8^a^	4.0^a^	30.7^a^	29.2^a^	8.6	8.7
Jang	2014	R,S	89	38	49.1	54.7	3.0^a^	2.5^a^	24.3^a^	25.3^a^	7.8	7.5
Wang	2015	R,S	81	135	61.2	63.5	3.8^a^	3.6^a^	26.2^b^	26.1^b^	8.3^b^	8.1^b^
Gu	2018	R,S	96	96	51^b^	50^b^	4.8^b^	4.8^b^	25.9^b^	25.6^b^	8^b^	8^b^
Deng	2020	R,S	58	58	52.0	50.6	5.0	4.9	NA	NA	8^b^	8^b^
Alimi	2018	P,M	50	50	NA	NA	3.9	4.1	NA	NA	7.5^a^	7.8^a^
Zhang	2020	R,S	62	62	47	46	NA	NA	26	25	9^b^	8^b^

*P*, prospective; *S*, single center; *R*, retrospective; *NA*, not avaliable; *BMI*, body mass index

^a^ Mean

^b^ Median M multiple.

### Operating time

**Figure 2:**
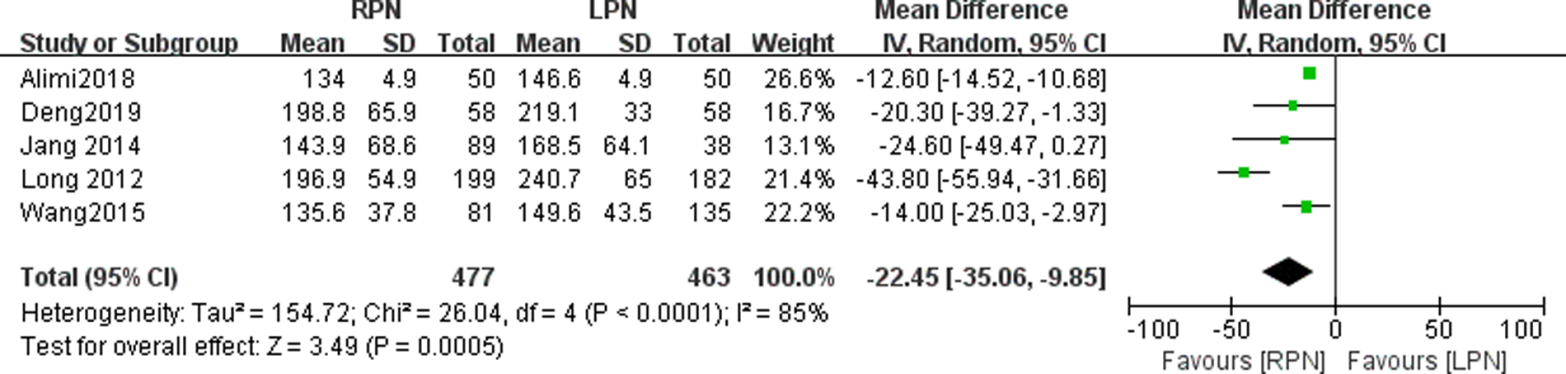
Forest plot for operating time between the RPN and LPN.

Our study showed that there was significant difference in operating time between the RPN and LPN groups (*n* = 940; 477 patients in the RPN group and 463 patients in LPN group; WMD: −22.45; 95% CI: −35.06 to −9.85; *I*^2^ = 85%; *p* = 0.0005; random-effects model; Figure 2).

### Estimated blood loss

**Figure 3:**
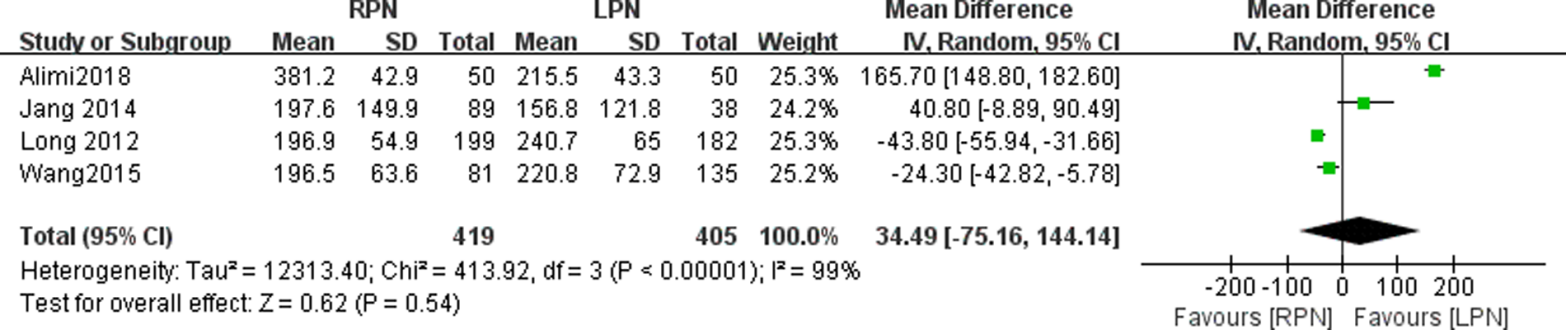
Forest plot for estimated blood loss between the RPN and LPN.

The estimated blood loss data were available in four studies. No statistically significant difference was found in the estimated blood loss between the RPN and LPN groups (*n* = 824; 419 patients in the RPN group and 405 patients in the LPN group; WMD: 34.49; 95% CI: −75.16–144.14; *p* = 0.54; I^2 ^= 99%; random-effects model; Figure 3).

### Warm ischemia time

**Figure 4:**
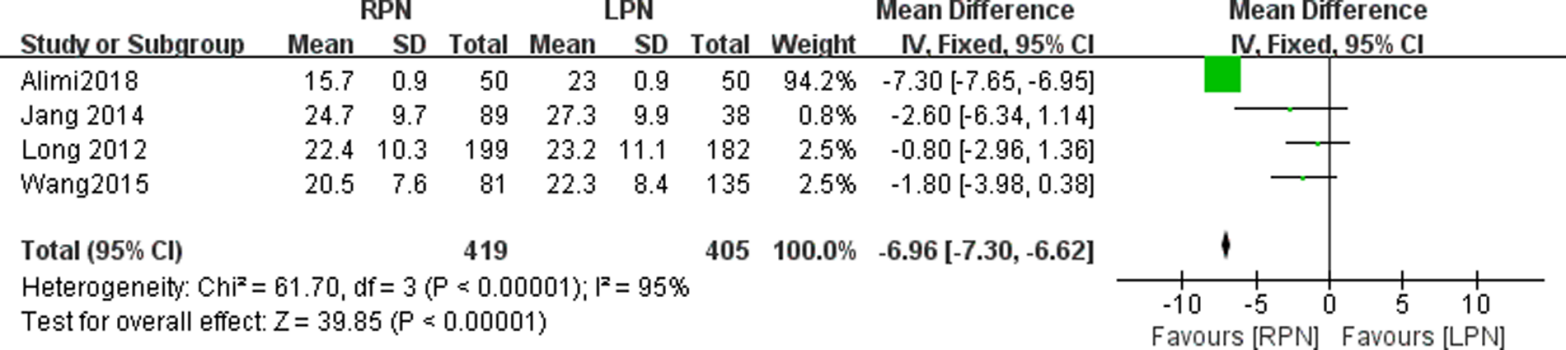
Forest plot for warm ischemia time between the RPN and LPN.

The warm ischemia time data were available in four studies. A statistically significant difference was found in the warm ischemia time between the RPN and LPN groups (*n* = 824; 419 patients in the RPN group and 405 patients in the LPN group; WMD: −6.96; 95% CI: −7.30–-6.62; *p* < 0.0001; *I*^2 ^= 0; random-effects model; Figure 4).

### Transfusion

**Figure 5:**
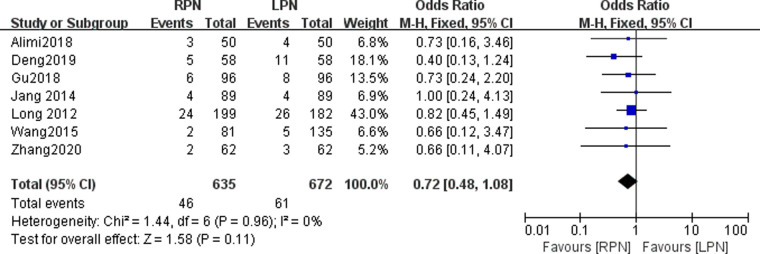
Forest plot for transfusion between the RPN and LPN.

Seven studies reported the transfusion in our meta-analysis. No statistically significant difference was found in the transfusion between the RPN and LPN groups (*n* = 1307; 635 patients in the RPN group and 672 patients in the LPN group; OR: 0.72; 95% CI: 0.48–1.08; *p* = 0.11; I^2^ = 0; fixed-effective model; Figure 5).

### Conversion

**Figure 6:**
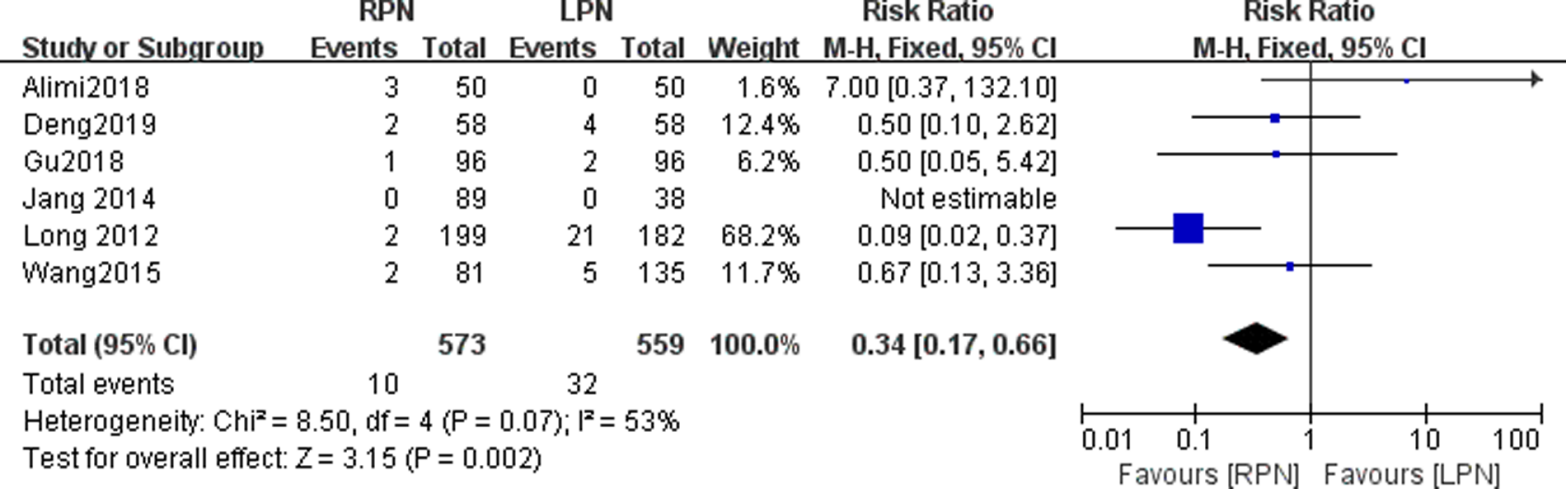
Forest plot for conversion between the RPN and LPN.

The pooled data showed a statistically significant difference between the RPN and LPN groups (*n* = 1132; OR: 0.34; 95% CI: 0.17 to 0.66; *I*^2 ^= 53%; *p* = 0.002; fixed-effects model; Figure 6).

### Hospital stay

**Figure 7:**
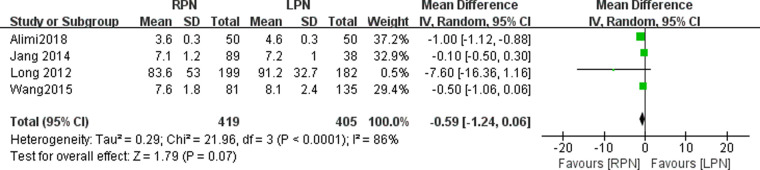
Forest plot for hospital stay between the RPN and LPN.

Four studies reported hospital stay data in this study. No statistically significant difference was found in the hospital stay between the RPN and LPN groups (*n* = 824; 419 patients in the RPN group and 405 patients in the LPN group; WMD: −0.59; 95% CI: −1.24–0.06; *p* = 0.07; *I*^2 ^= 86%; random-effects model; Figure 7).

### Intraoperative complications

**Figure 8:**
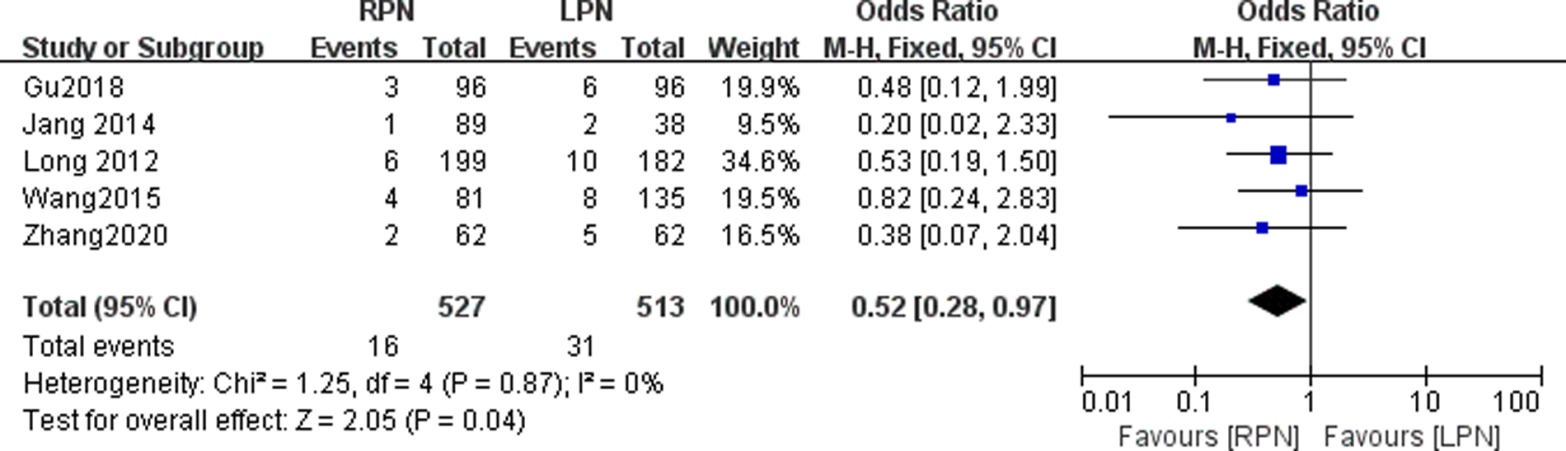
Forest plot for intraoperative complications between the RPN and LPN.

Five studies reported the intraoperative complications. A statistically significant difference was found in the intraoperative complications between the RPN and LPN groups (*n* = 1040; 527 patients in the RPN group and 513 patients in the LPN group; OR: 0.52; 95% CI: 0.28–0.97; *p* = 0.04; I^2 ^= 0; fixed-effects model; Figure 8).

### Postoperative complications

**Figure 9:**
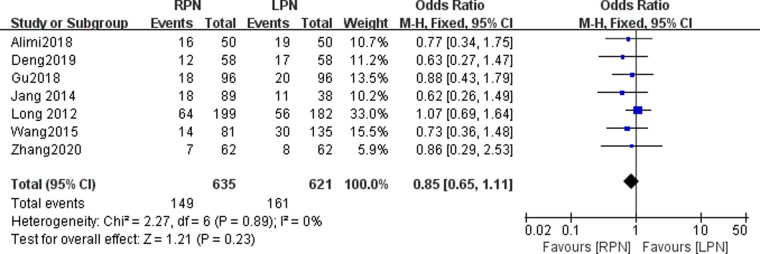
Forest plot for postoperative complications between the RPN and LPN.

Data on postoperative complications were available in seven studies. No statistically significant difference was found in the postoperative complications between the RPN and LPN groups (*n* = 1256; 635 patients in the RPN group and 621 patients in the LPN group; OR: 0.85; 95% CI: 0.65–1.11; *p* = 0.23; *I*^2 ^= 0; fixed-effective model; Figure 9).

### Postoperative renal function

**Figure 10:**
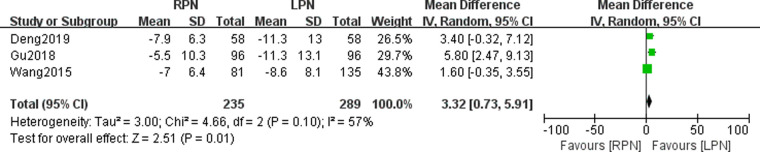
Forest plot for postoperative renal function between the RPN and LPN.

Three studies were included in our meta-analysis to pool the postoperative renal function. A statistically significant difference was found in the postoperative function between the RPN and LPN groups (*n* = 524; 235 patients in the RPN group and 289 patients in the LPN group; WMD: 3.32; 95% CI: 0.73–5.91; *p* = 0.01; *I*^2^ = 57; random-effects model; Figure 10).

### Positive surgical margin

**Figure 11:**
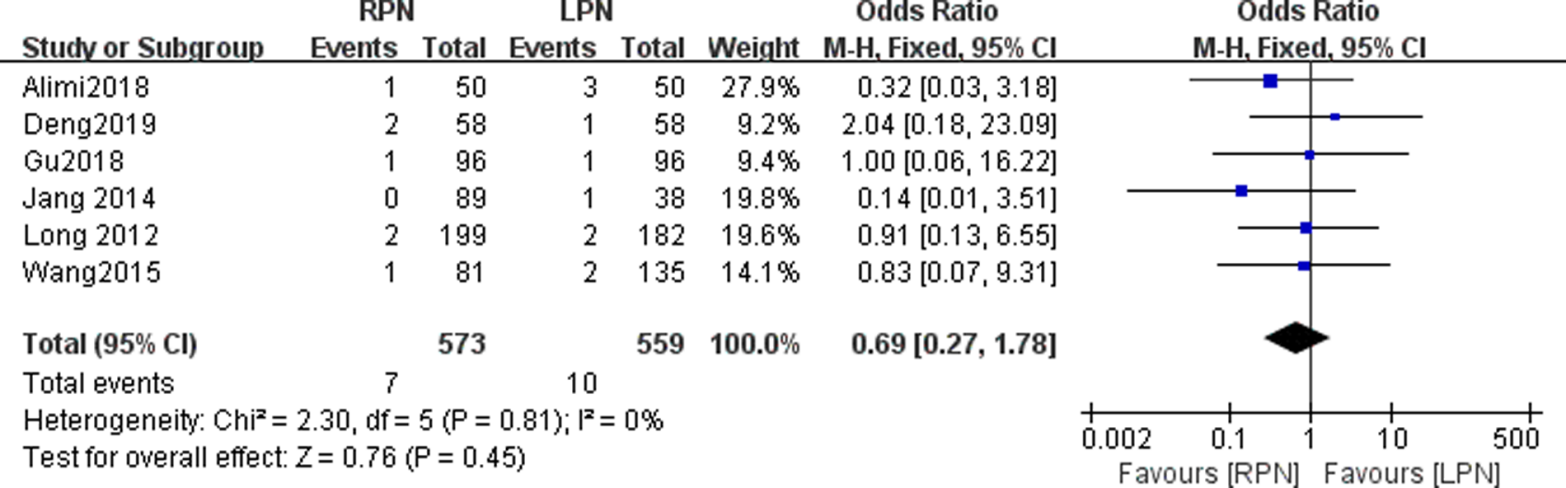
Forest plot for postive surgical margin between the RPN and LPN.

Six studies reported a positive surgical margin. No statistically significant difference was found in the positive surgical margin between the RPN and LPN groups (*n* = 1132; 573 patients in the RPN group and 559 patients in the LPN group; OR: 0.69; 95% CI: 0.27–1.78; *p* = 0.45; *I*^2 ^= 0; fixed-effects model; Figure 11).

### Major postoperative complications

**Figure 12:**
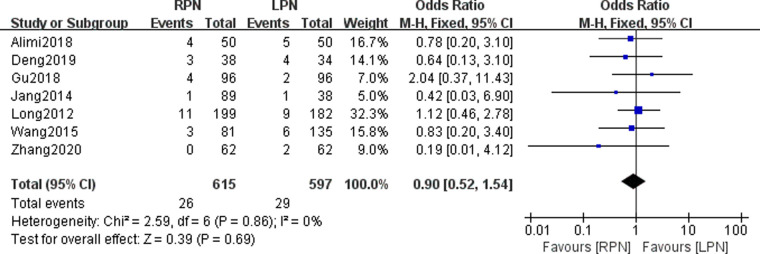
Forest plot for major postoperative complications between the RPN and LPN.

Seven studies were included in our meta-analysis to pool the major postoperative complications. No statistically significant difference was found in the major postoperative complications between the RPN and LPN groups (*n* = 1212; 615 patients in the RPN group and 597 patients in the LPN group; OR: 0.90; 95% CI: 0.52–1.54; *p* = 0.69; *I*^2 ^= 0; fixed-effects model; Figure 12).

## Discussion

In our study, we found no significant difference in the estimated blood loss, hospital stay, postoperative complication rate, positive surgical margin and transfusion between the RPN and LPN groups. The postoperative renal function, operating time, conversion rate to radical nephrectomy and warm ischemia time were lower in the RPN group than in the LPN group. In our meta-analysis, the pooled data of warm ischemia time indicated a shorter warm ischemia time in the RPN group than in the LPN group. Choi et al. reported a similar outcome to ours (*p* = 0.005) (16).

Regarding the conversion rate to radical nephrectomy, our meta-analysis found that the RPN group had a lower conversion rate than the LPN group. However, Aboumarzouk et al. conducted a meta-analysis including 717 patients and found that the conversion rate was not significantly different between the RPN and LPN groups (*p* = 0.84). The cause may be due to different baseline characteristics in different studies. This outcome was consistent with previous studies (19, 21). This showed that the safety of RPN and LPN were equival for anatomically complex renal tumors.

In our meta-analysis, the patients in the RPN group showed better recovery in the postoperative renal function than those in the LPN group. The RPN group showed a low warm ischemia time and a satisfactory postoperative eGFR rate. The postoperative renal function was thought to be associated with the duration of warm ischemia time. When the warm ischemia time was >30 min, the postoperative eGFR rate decreased (24). Overall functional reduction was similar in both RPN and LPN groups, which may have been misperceived because of the compensation of the contralateral kidney.

In our study, the warm ischemia time was lower in the RPN group than in the LPN group, causing quick renal function recovery. The cause may be attributed to using precise handling instruments, three-dimensional magnified vision and precise dissection of the renal pedicle, and better conducted tumor resection with robotic assistance. Kopp et al. performed a study to analyze the related factors associated with postoperative renal function after partial nephrectomy. They found that the RENAL score could predict the estimated glomerular filtration rate and warm ischemia time (25). Simone and Brassetti also found totally off-clamp RPN seemed to be equal to treat complex renal masses compared to on-clamp surgical method (7, 26, 27). And, Ferriero et al. found that the effect of learning curve on outcomes of off-clamp RPN could be negligible after a proper training in minimally invasive surgery (28).In our meta-analysis, the pooled data of the warm ischemia time showed high heterogeneity. This finding may be related to the tumor location and surgeons with different surgical skills. Recently, Bertolo et al. reported a study indicating that different reconstruction methods could shorten the ischemia and operating times (29). The different suture skills may be the causes of high heterogeneity. Daniel et al. found that prolonged warm ischemia time is related with worse perioperative outcomes (30). However, a comparative study performed by Homayoun et al. found that the prolonged warm ischemia time associated needs to be mitigated in RPN (31). We also believe that the advantage of RPN is not the significant progress in WIT or operative time, but rather the broadened indications of minimally invasive partial nephrectomy.

In our meta-analysis, we found that the operative time was shorter in the RPN group than in the LPN group. Choi et al. performed a meta-analysis comparing RPN and LPN to treat renal tumors (16). They found no significant difference between the two groups. This finding was not consistent with our study findings.

We found that intraoperative complications were lower in the RPN group than in the LPN group. However, Zhang et al. found no significant difference between the groups (*p* = 0.78) (22). Zhang et al. performed a meta-analysis and found that the intraoperative complications showed no statistically significant difference between the groups (22). In our study, we included patients with a RENAL nephrometry score ≥7, which may explain the difference with Zhang's study. Additionally, different surgeons have different surgical skill levels for RPN or LPN.

Our meta-analysis also found that the positive surgical margin showed no statistically significant difference between the RPN and LPN groups (*p* = 0.45). Similarly, Zhang et al. performed a meta-analysis and found no statistically significant difference between the RPN and LPN groups (*p* = 0.61). Aboumarzouk et al. also reported a similar outcome (*p* = 0.93). This may indicate that the resected parenchymal volume was smaller in the RPN group compared with LPN. Additionally, age, different tumor locations, simple enucleation and low-grade tumor were found to be independent factors of PSMs (32).

In our meta-analysis, the estimated blood loss showed no statistically significant difference between the RPN and LPN group. Zhang et al. also found that the estimated blood loss exhibited no statistically significant difference between the groups (*p* = 0.75). This finding is consistent with our study findings. However, the high heterogeneity in the estimated blood loss was likely due to the difference in familiarity of surgeons to the surgical process. However, Chang et al. also performed a propensity-score-matching study and found that RPN resulted in a significantly lower mean estimated blood loss than LPN (*p* = 0.025) (33). Several systematic reviews and meta-analyses reported similar outcomes (14, 16, 34).

In our study, we reported that a statistically significant difference was found in the intraoperative complications between the RPN and LPN groups (*p* = 0.04). Similarly, Cacciamani et al. performed a meta-analysis found that RPN was superior for intraoperative complications (35). However, Gu et al. conducted a propensity score-based analysis indicated that no statistically significant difference was found between RPN and LPN groups (10). We believed that the results about overall complications in the presented analysis were similar to those in other studies.

### Limitations

Our study had several limitations. First, we did not include RCTs. This can lower the evidence of our study. Second, the different studies reported variable tumor sizes which could increase the heterogeneity and lower the confidence of our meta-analysis and could affect the warm ischemia time and postoperative renal function. We did not adjust the common baseline characteristics of patients. And, we should also do a subgroup analysis at higher scores (7–8, 8–10. The different definitions of nomenclature and functional outcomes could lead to heterogeneity (36). Third, we did not evaluate the oncological outcomes regarding overall survival, recurrence-free survival and cancer-specific survival. In our meta-analysis, some studies did not perform propensity score-based analysis, a finding that could increase the heterogeneity. Alimi et al. conducted a multicenter study involving different surgeons that also increased the heterogeneity. Regarding high heterogeneity, we did not conduct sensitivity analysis or subgroup analysis. We also did not identify the causes of high heterogeneity. We compared the perioperative outcomes and postoperative renal function outcomes.

## Conclusion

Our meta-analysis showed that RPN could achieve comparable outcomes in the estimated blood loss, hospital stay, operating time, positive surgical margin and transfusion. RPN achieves better outcomes in the postoperative renal function, warm ischemia time, conversion rate to radical nephrectomy and intraoperative complications. More RCTs should be performed to clarify the effectiveness of RPN and LPN.

## Data Availability

The original contributions presented in the study are included in the article/Supplementary Material, further inquiries can be directed to the corresponding author.
